# Wearable Sensing of In-Ear Pressure for Heart Rate Monitoring with a Piezoelectric Sensor

**DOI:** 10.3390/s150923402

**Published:** 2015-09-16

**Authors:** Jang-Ho Park, Dae-Geun Jang, Jung Wook Park, Se-Kyoung Youm

**Affiliations:** 1 Medical IT Convergence Research Center, Korea Electronics Technology Institute, Seongnam 463-816, Korea; E-Mail: janghopark@keti.re.kr; 2 Samsung Advanced Institute of Technology, Samsung Electronics Co. Ltd., Suwon 443-803, Korea; 3 Creative Innovation Center, LG Electronics Co. Ltd., Seoul 137-893, Korea; E-Mail: ubihuman@gmail.com; 4 Industry-Academic Cooperation Foundation, Dongguk University, Seoul 100-715, Korea; E-Mail: sekyoungyoum@gmail.com

**Keywords:** wearable heart rate monitoring, in-ear pressure variance, piezoelectric sensor

## Abstract

In this study, we developed a novel heart rate (HR) monitoring approach in which we measure the pressure variance of the surface of the ear canal. A scissor-shaped apparatus equipped with a piezoelectric film sensor and a hardware circuit module was designed for high wearability and to obtain stable measurement. In the proposed device, the film sensor converts in-ear pulse waves (EPW) into electrical current, and the circuit module enhances the EPW and suppresses noise. A real-time algorithm embedded in the circuit module performs morphological conversions to make the EPW more distinct and knowledge-based rules are used to detect EPW peaks. In a clinical experiment conducted using a reference electrocardiogram (ECG) device, EPW and ECG were concurrently recorded from 58 healthy subjects. The EPW intervals between successive peaks and their corresponding ECG intervals were then compared to each other. Promising results were obtained from the samples, specifically, a sensitivity of 97.25%, positive predictive value of 97.17%, and mean absolute difference of 0.62. Thus, highly accurate HR was obtained from in-ear pressure variance. Consequently, we believe that our proposed approach could be used to monitor vital signs and also utilized in diverse applications in the near future.

## 1. Introduction

Cardiovascular disease (CVD), which includes hypertension, myocardial infarction, coronary artery diseases, and stroke, has become a serious problem and is currently one of the major causes of disability and death globally. In fact, more than 17 million people worldwide die each year because of CVD, and it accounts for about 30% of all deaths per year [1]. While many of the risk factors for CVD are known, such as tobacco use, physical inactivity, obesity, and diabetes, resting heart rate (HR) is one of the simplest cardiovascular parameters and an independent risk factor [2]. Resting HR has prognostic importance in that an elevated resting HR is strongly related with mortality in the general population, the elderly, and patients with myocardial infarction, congestive heart failure, diabetes, or hypertension [2]. Thus, frequent HR monitoring in everyday life is necessary to assess and prevent cardiovascular problems in advance.

Vital signs such as HR, respiration, and blood pressure were in the past possible to measure by medical systems in hospitals. However, recent technological advances in physiological sensors, processing capabilities, and wireless communications have enabled individuals to monitor their health status outside of hospitals via wearable biosensor systems [3]. Compared with previous medical devices, wearable sensor systems provide nonintrusive solutions for long-term monitoring without constraints on time and place. A further advantage is that such systems can offer real-time feedback about their health status to the patients themselves or even a professional physician at a hospital [4]. Therefore, wearable HR monitoring systems play an important role in the monitoring of cardiovascular exacerbations in the early stage, during catastrophes that may occur with high risk individuals, or during emergency situations [5].

Many technologies and methodologies have been introduced for wearable HR monitoring. Among these technologies, those that monitor photoplethysmogram (PPG), which is obtained by optical detection of blood volume changes in the microvascular bed of the tissue using light-emitting diodes and photodetectors, are the most widely utilized. PPG sensors have mainly been applied to fingers, earlobes, or wrists in the form of tweezers- or watch-shaped apparatuses [6]. Also, a customized ear mold equipped with a reflective PPG sensor has been applied to the ear channel [7]. Conventional measurement of electrocardiogram (ECG), which is the gold-standard method for HR monitoring, is not appropriate for wearable sensing because of the adhesive electrodes and wires utilized. However, recent studies have proposed and investigated T-shirts and band type wearable ECG systems that use conductive fabric, flexible printed circuit boards, or textile circuits [8]. In addition, stethoscopes that use a microphone to measure phonocardiography have also been proposed as wearable and portable devices [9].

However, despite significant progress in wearable systems, various issues that impede extensive application have been reported. These issues include bulky sensors, connections to additional hardware modules, skin irritation, and laundry [9]. In order to solve these problems, Teichmann *et al.* recently presented a bendable and flexible device that can be placed in a shirt pocket using noncontact sensor principles [10]. However, very few studies have presented a high acceptance rate by users, and there are still many challenges that have to be resolved in order for wearable systems to become accepted by normal people and patients [3,8]. For examples, existing methods have limitations to utilization in particular situations when the user is running or swimming in which hands and feet are excessively moving. The human head is rather the least moving and least affected part by strenuous exercise or movement according to circumstances. Thus, new methods or alternatives are required for vigorous follow-up studies and applications.

In this paper, we present our proposed wearable HR monitoring sensor device developed based on pressure variance in the ear canal surface. The ear is an excellent site for users to accept a wearable device. In addition, the ear canal is partially composed of cartilage and bone and located in the temporal bone, thus providing the proper conditions for anchoring the sensor apparatus [11]. The ensuing sections present the physiological principle that enables HR monitoring from the ear canal surface, the material comprising the sensor, design and implementation of the wearable sensor device, the verification process and the results obtained, and our conclusions.

## 2. Physiological Background and Piezoelectric Sensor

When the left ventricle of the heart rhythmically contracts, blood is ejected into the arterial system, and the elasticity of the arteries enable a pulse wave to be generated and propagated. The pulse wave observed in the arterial system is the summation of a forward (incident) wave and a backward wave [12]. The forward wave travels from the heart toward the periphery, whereas the backward wave is caused by the forward wave’s reflection within the peripheral arterial system [12]. The characteristics of the arterial pulse wave differ slightly according to the sites at which the measurements are taken because of the complicated topology of the arteries. However, in general, all arterial pulse waves present the general function of the cardiovascular system: the systolic function of the left ventricle [13].

The carotid artery, which has diameter in the range 6.1 to 6.5 mm, originates along the aortic arch and travels upward to supply oxygenated blood to the brain. The carotid arterial pressure and diameter change linearly as blood flows, and the hemodynamic change generates the ascending aortic pressure waveform around the artery [14,15]. Ultrasound with the Doppler technique is the gold-standard method for observing volume changes in the carotid artery, whereas a tonometry attached to the subject’s neck is also typically utilized to measure the pressure wave propagated to the skin surface [16].

**Figure 1:**
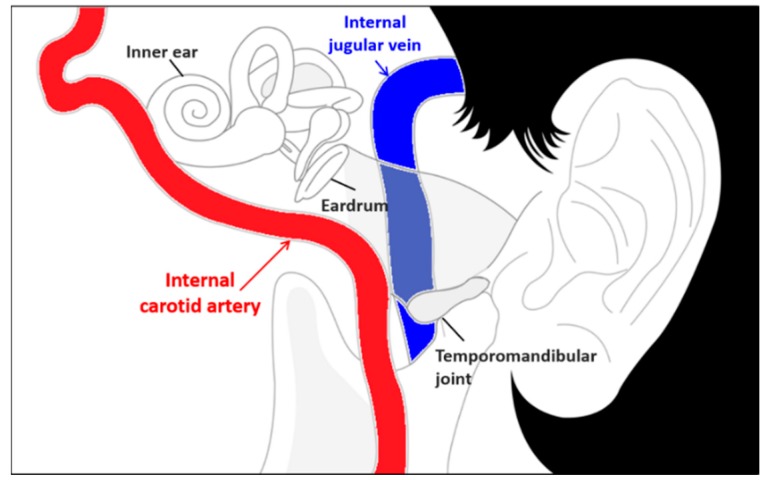
Anatomical view of the vessels around the ear.

As illustrated in the anatomical view depicted in Figure 1, the internal carotid artery, which arises from the carotid artery, is located near the ear. The internal carotid artery also has a similar characteristic; that is, it expands and propagates a pressure wave to the ear canal surface during the inrush of blood. Consequently, it has been hypothesized that pressure variance measured from the ear canal surface has a consistent pulse waveform, and the in-ear pulse wave (EPW) presents sharp upstrokes reflective of heartbeats.

The ear canal is approximately 2.5 cm long and has a diameter of about 0.7 cm. The surface of the skin in the ear canal is one of the most sensitive parts of the human body. Any sensor material that is intended to be utilized there therefore has to be small and soft, which are properties possessed by the piezoelectric sensor used in this application. The sensor used is flexible, lightweight, and is composed of mechanically durable plastic film with the unique ability of being configurable into various shapes and sizes. Piezoelectric materials convert strain or stress into electrical energy when they are mechanically deformed [17]. They operate over a wide temperature range and have a high frequency response.

Piezoelectric sensors are widely used in medical applications such as patient monitoring and human motion analysis. In particular, a piezoelectric sensor can be ideally utilized in harsh environments and is suitable for measuring tiny pressure variances under high static pressure [18]. This property raises the possibility of successful monitoring in our application, because the pressure for anchoring the film sensor to the ear canal surface is high and static, and the pressure variance from a heartbeat is relatively low. In this study, we utilized a customizable piezoelectric film sensor sheet manufactured by Measurement Specialties [19]. The film sensor sheet is metallized with silver ink which enables optimized applications where mechanical stress is being applied.

## 3. In-Ear Pressure Sensing Device

### 3.1. Apparatus Design Concepts

**Figure 2:**
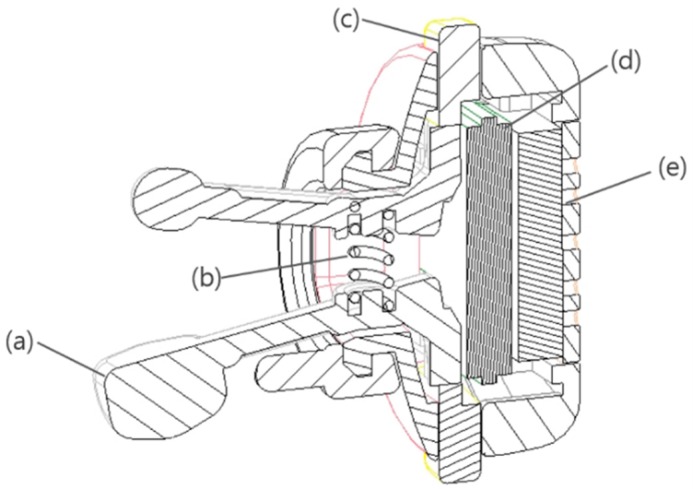
Structure of the in-ear pressure sensing device: (**a**) stick covered with piezoelectric film sensor; (**b**) spring to push both sticks in different directions; (**c**) button to control the position of both sticks; (**d**) hardware circuit module; (**e**) coin-cell battery.

A specialized design is required for any apparatus specified to measure pressure variance from the ear canal surface accurately. High wearability should also be guaranteed to facilitate long-term use in everyday life. Further, to enable accurate measurement, the piezoelectric film sensor needs to be sufficiently adhered to the skin surface of the external ear canal. When these conditions are satisfied EPW can be consistently converted to electrical current and various noises, caused by the user’s movements and activities, can be minimized. For a comfortable fit, the apparatus should also not cause the user any pain, and should be lightweight in order for the user to wear it for hours. Furthermore, it should not interfere with the user’s hearing as an insert-type device in the ear. To meet these requirements and considerations, we designed a scissor-shaped apparatus with the structure illustrated in Figure 2.

**Figure 3:**
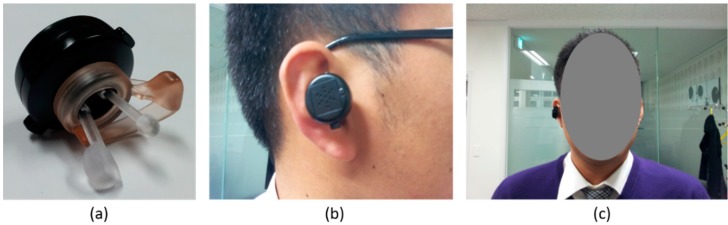
(**a**) In-ear pressure sensing device prototype; (**b**) wearing example (lateral view); (**c**) wearing example (frontal view).

We cut the piezoelectric film sheet into a 3.5 mm × 3.5 mm sized piece that can covers the head part of a longer stick. The outside surface (signal) and the inside surface (ground) of a piezoelectric sensor were wired with shielded cable respectively to protect EPW signal and minimize the noise. When the user wears the apparatus, the two sticks are inserted into the ear canal, and a spring enables proper extension between the sticks. Thus, the head parts of both sticks are able to remain adhered to opposite sides of the ear canal surface. The head positions that make contact with the ear canal surface can be changed by pushing and rotating buttons. Because the optimal point on the ear canal surface for EPW sensing varies slightly from individual to individual, the best point for a particular individual can be found by varying the stick positions. The hardware circuit module and coin-cell battery are located in the body of the apparatus. Figure 3 shows the appearance of a prototype manufactured according to the proposed design along with examples of it being worn.

### 3.2. Hardware Circuit Module

**Figure 4:**
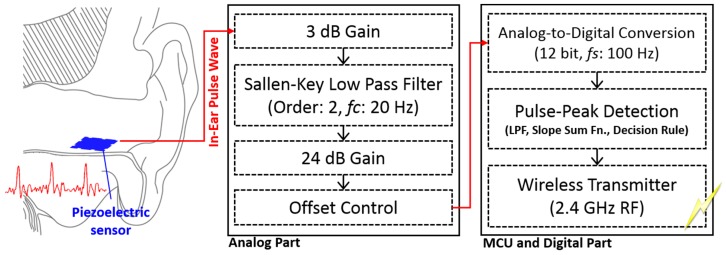
Circuit module and signal processing flow.

Electrical current, generated from the piezoelectric sensor, is converted to voltage. Then, it is 3 dB pre-amplified and passed to a buffer to prevent loading effect. A second-order low-pass filter (LPF) with a cut-off frequency (*fc*) of 12 Hz is used to remove the high frequency noises [20]. The smoothed signal is then amplified to a maximum of 24 dB, and an offset control circuit adds DC voltage to the amplified signal. Consequently, the final output signal of the analog part has a voltage range of 0 to +3.3 V, which can be appropriately processed in the digital part (Figure 4).

**Figure 5:**
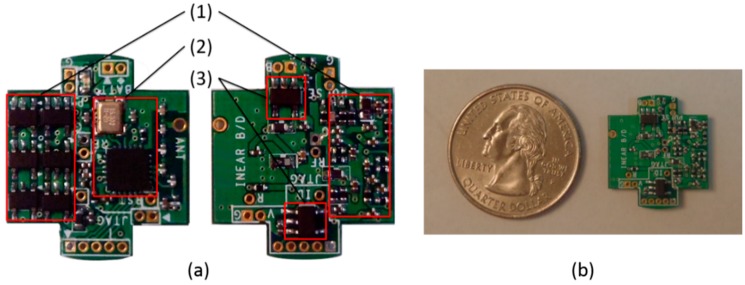
Circuit board (15 × 17 mm): (**a**) top and bottom views of circuit board, (1) analog part, (2) MCU and digital part, (3) power regulator; (**b**) circuit board size compared to 25 cent coin.

The digital circuit comprises a micro-controller unit (MCU) and a wireless communication module with an antenna. Analog EPW is converted to a 12-bit digital signal at a sampling frequency (*fs*) of 100 Hz by an analog-to-digital converter (ADC) module in the MCU. A software program embedded in the MCU detects pulse peaks in real time, and then the HR value is periodically transmitted to the host via a wireless communication module. The module utilizes radio frequency (RF) on the 2.4 GHz band, and the RF and antenna power output specifications are 0 dB and 0.5 dB, respectively (Figure 4). The complete circuit module is depicted in Figure 5. It is designed and implemented in the shape of a cross to utilize space efficiently and to have a minimal size to facilitate insertion into the proposed apparatus.

### 3.3. Pulse-Peak Detection

The pulse peak detection algorithm comprises two stages. The first stage is morphological conversion, in which the raw EPW is converted to a more robust signal that is impulsive and distinct [21]. The second stage is application of knowledge-based rules to detect pulse peaks. The algorithm is implemented in the C language in an embedded software environment, and runs in real time.

**Figure 6:**
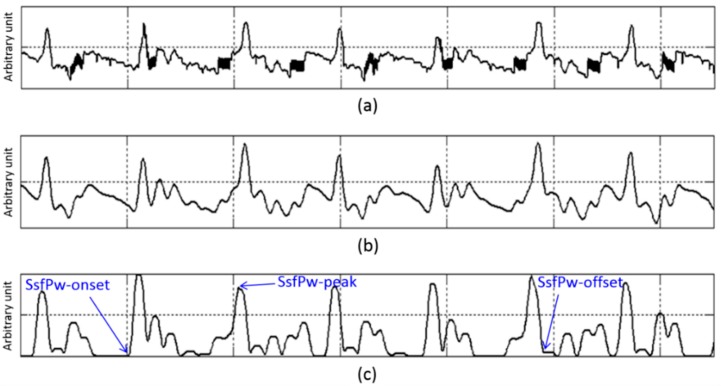
In-ear pulse wave recorded for 6.5 s: (**a**) raw EPW; (**b**) smoothed EPW; (**c**) converted (SSF) EPW.

The morphological conversion stage comprises three phases: (1) finite impulse response (FIR) LPF; (2) differentiation; and (3) slope sum function (SSF). An FIR filter is employed to suppress high frequency factors by muscle influence and 50/60 Hz noise interference. A constrained equiripple FIR filter (window size: 500 ms, *fc*: 8 Hz) was designed and applied. This filter guarantees that the stop-band of the filter begins at a specific frequency and provides the desired minimum stop-band attenuation [22]. Then, differentiation with half-rectification is applied to enhance the ascending part and suppress the descending part of the EPW. The SSF, a signal transformation scheme, is applied, for the same purpose as the differentiation process (Figure 6). Window size of the SSF is set as 128 ms because the ascending portion of a typical pulse wave is 128 ms [23].

**Figure 7:**
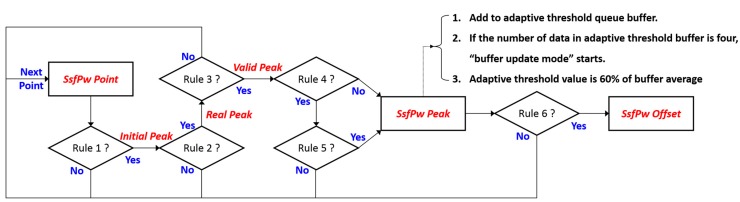
Decision flow of EPW peak detection.

**Figure 8:**
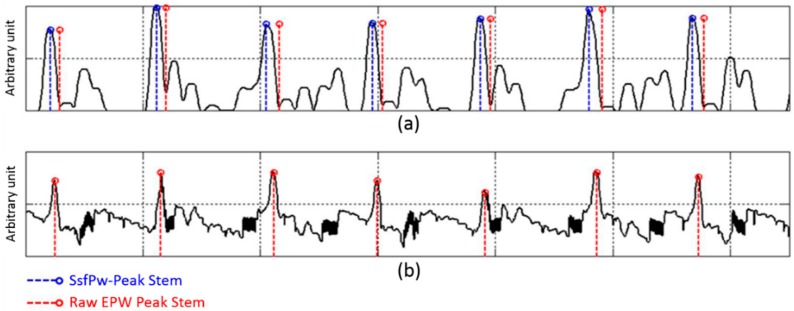
SsfPw peak and raw EPW peak detected via decision rules: (**a**) SsfPw with its peaks and offsets; (**b**) raw EPW with its peaks.

The offset point of the EPW that passes the SSF (SsfPw) corresponds with the peak of the raw EPW. The decision rules therefore aim to detect the peak and offset point of SsfPw. Figure 7 and Table 1 describe the knowledge-based decision process used to detect EPW peak. An example of the peak detection result is shown in Figure 8.

**Table 1:** Decision rules for SsfPw-offset detection.

Decision Rule	Description
# 1	Are both the previous and next points lower than the current point?
# 2	Is it continuously descending until 50% of the “*Initial Peak*” value?
# 3	Is the distance between any continuous “*Real Peaks*” further than 250 ms?
# 4	Is it in “update mode”?
# 5	Is “*Valid Peak*” higher than the adaptive threshold?
# 6	Find the minimum point from “*Valid Peak*” within 150 ms in the forward direction.

## 4. Validity Study

### 4.1. Study Design

To evaluate the validity of our developed monitoring device, a comparative analysis was performed based on clinical data with reference equipment. A total of 58 healthy volunteers aged 26–44 years participated in our clinical experiment (Table 2). Each subject was informed of the procedure and the purpose of clinical data acquisition prior to the experiment, and subsequently signed a participation agreement form. None of the participants had any self-reported cardiovascular disease such as hypertension, cardiac infarction, or arrhythmia.

**Table 2:** Participants’ personal information.

	N	Age	Height (cm)	Weight (kg)
Male	43	36.3 ± 5.6	172.9 ± 4.9	72.5 ± 11.6
Female	15	31.7 ± 5.3	163.2 ± 3.7	53.5 ± 7.1
Total	58	35.3 ± 5.8	170.6 ± 6.3	67.8 ± 13.4

A commercial device (ECG-KIT V3.0, PhysioLab Co., Ltd, Busan, Korea) for the ECG measurement was utilized as reference equipment [24]. The device measures ECG using three wired leads in contact with the user’s right arm, left arm, and right leg, respectively. ECG-KIT includes instrumentation amplifier and filters (LPF, HPF, and notch), and provides an analog waveform as a final output. We connected its analog output to the ADC input port of the MCU in our developed in-ear device for the experimental setting. Thus, both analog signals, EPW and ECG, were simultaneously converted to digital signals with the same *fs* (100 Hz) and ADC resolution (12 bit). This setting was intended to secure time-synchronized measurement between our targeted and reference signals.

Each subject wore the in-ear device and ECG electrodes while sitting in a relaxed position, and EPW and ECG were simultaneously measured. A rest time of 5 min was given before the measurement in order to induce a stable state in each subject. Data were recorded for 30 s from each subject.

### 4.2. Comparative Parameter

Each dataset obtained comprised EPW and ECG data simultaneously recorded for 30 s. Thus, a total of 58 datasets were obtained through the clinical experiment with the 58 subjects. Physiologically, an EPW peak and an ECG R-peak cannot occur at the same time owing to the pulse transit time [25]. Therefore, peak-to-peak interval was chosen as the parameter to use for comparison of the EPW and ECG signals. Each interval was calculated as

**Figure 9:**
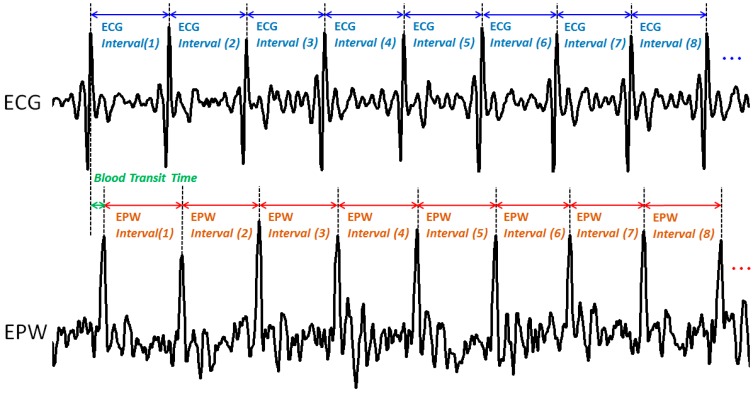
Concurrently recorded ECG and EPW signals, and their peak-to-peak intervals for comparative analysis.

(1) Interval(n)=No. of data samples between successive peaks

and extracted from EPW and ECG, respectively (Figure 9). The EPW peak was automatically determined by the EPW peak detection algorithm outlined in Section 3.3. Conversely, the ECG R-peak was manually annotated by three examiners. Each examiner marked the ECG R-peak position (index) using MATLAB (Matrix Laboratory, USA) Ver. 2009, and unconformable peak positions were modified with the consent of all three examiners.

An ECG R-peak occurs when the left ventricle contracts to pump blood out, whereas an EPW peak occurs when the pulse wave is generated in the internal carotid artery near the ear canal [25]. In that typical pulse transit time from heart to arteries near the ear, ranging from 10 to 20 ms [26], an EPW peak corresponding to an ECG R-peak occurs right after the ECG R-peak occurs in the concurrently recorded plot. In this way, intervals 20–23 numbers of ECG_*Interval*_ (*n*) and their corresponding EPW_*Interval*_ (*n*) were extracted from each dataset.

### 4.3. Evaluation

Interval differences between ECG and EPW were investigated to determine the validity of our proposed sensor device. To this end, four quantitative measures were utilized: sensitivity, positive predictive value (PPV), mean absolute difference (MAD), and normalized error rate (ER_*Norm*_). These measures are defined in Equations (2) to (5), respectively.

(2) $$Sensitivity\;\left(\%\right)=\frac{TP}{TP+FN}\;\times 100\;\left(\%\right)$$

(3) $$PPV\;\left(\%\right)=\frac{TP}{TP+FP}\;\times 100\;\left(\%\right)$$

(4) $$MAD\;\left(samples\right)=\frac{1}{N}\;\overset{N}{\underset{n=1}{\sum }}\left|ECG_{Interval}\left(n\right)-\;EPW_{Interval}\left(n\right)\right|$$

(5) $$ER_{Norm}\;\left(\%\right)=\frac{\sum_{n=1}^{N}\left|ECG_{Interval}\left(n\right)-\;EPW_{Interval}\left(n\right)\right|}{\sum_{n=1}^{N}ECG_{Interval}\left(n\right)}\;\times 100\;\left(\%\right)$$

Peak-to-peak interval of ECG or PPG is used to HR monitoring or HRV (heart rate variability) analysis. In HR monitoring, 20 ms interval error possibly cause maximum error ranges of 0.85–3.23 bpm. However, HR is an integer value and calculated from the averaged interval. HR error is therefore much less than the maximum error range. In HRV analysis, an indicator that utilizes time delay is the pNN*x*; the percentage of NN intervals in a 24-h time series differing by more than *x* ms [27]. The pNN50 or pNN20 is typically utilized [27]. Thus, maximum interval error of 20 ms is allowed to HRV analysis in time domain. For these reasons, we defined true detection as the difference between ECG_*Interval*_ (*n*) and its corresponding EPW_*Interval*_ (*n*) being less than or equal to two samples (20 ms). Thereby, true positive (TP), false positive (FP), and false negative (FN) are defined as follows:

TP—the number of EPW_*Interval*_ (*n*) that satisfy the true detection condition,
FP—the number of EPW_*Interval*_ (*n*) that do not satisfy the true detection condition,
FN—the number of ECG_*Interval*_ (*n*) that do not satisfy the true detection condition.

Accordingly, sensitivity represents the proportion of “the number of truly detected intervals” out of the “total number of ECG_*Interval*_ (*n*),” whereas PPV signifies the proportion of “the number of truly detected intervals” out of the “total number of EPW_*Interval*_ (*n*).” [28] MAD and ER_*Norm*_ represent the interval difference per unit interval and unit sample, respectively. In addition, we adopted the Bland-Altman analysis to assess the agreement of EPW_*Interval*_ with ECG_*Interval*_ [29]. We utilized SPSS (Statistical Package for the Social Science, Armonk, NY, USA) Ver. 22 for statistical data processing.

## 5. Results and Discussion

Table 3 presents a summary of the acquired EPW data. Totals of 1297 of peaks, 1239 intervals, and 111,401 samples of interval length were detected from the 58 datasets, and their HR was 66.74 bpm on average. Average 22.36 ± 0.67 of peaks, 21.36 ± 0.67 of intervals, and 1920.71 ± 34.48 samples of interval length existed in each dataset, and its HR was 66.74 ± 1.82 on average. In general, the normal HR of an adult ranges from 60 to 100 bpm [30]. Thus, the HR of the subjects in this study was considered as relatively low, and presented in a narrow range: standard deviation (SD) of EPW-HR is 1.82. We surmise that this result was obtained because the subjects measured ECG and EPW in a very stable state, and belong to a similar age group.

**Table 3:** Summary of acquired EPW data.

	No. of Peaks	No. of Intervals	Interval Length (samples)	Heart Rate (bpm)
Dataset	22.36 ± 0.67	21.36 ± 0.67	1920.71 ± 34.48	66.74 ± 1.82
Total	1297	1239	111,401	66.74

Table 4 shows the performance evaluation results for the EPW device. TP, FP, and FN were 1204, 35, and 34, respectively. Sensitivity was 97.25%, and PPV was 97.18%. Even in the case where PPG and ECG were concurrently measured with standard devices and methods, the peak-to-peak interval of PPG was not perfectly in agreement with the corresponding peak-to-peak interval of ECG [31], because the physiological system of the human body has various, tiny and nonlinear effects on the blood flow [32]. We therefore regard our obtained sensitivity and the PPV as outstanding achievements, although two samples (20 ms) were set as allowable error.

**Table 4:** Performance evaluation results.

TP (No. of Intervals)	FP (No. of Intervals)		FN (No. of Intervals)
1204	35		34
Sensitivity (%)	PPV (%)	MAD (samples)	ER_*Norm*_ (%)
97.25	97.18	0.62	0.68

MAD was 0.62 samples, and ER_*Norm*_ was 0.68%. This means that EPW had on average 0.62 samples (6.2 ms) of error per unit interval, and on average 0.68% (68 µs) of error per unit sample, compared with ECG. These numerical values also support the accuracy and validity of our proposed device for HR monitoring.

**Figure 10:**
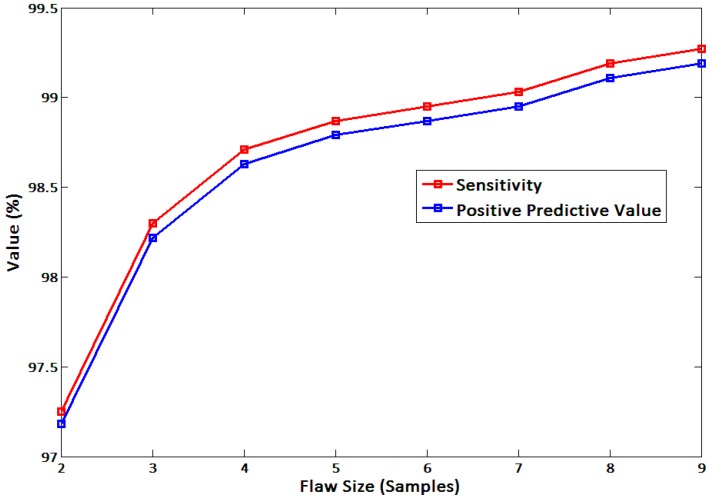
Sensitivity and PPV curves of the in-ear device.

As the allowable error (flaw size) increased, the sensitivity and the PPV naturally increased. Increasing curves are depicted in Figure 10 with increasing flaw size from two to nine. When the flaw size is greater than three, the sensitivity and the PPV are above 98%. When the flaw size is greater than eight, the sensitivity and the PPV are above 99%. In the case where the HR varies from 50 bpm to 100 bpm, flaw sizes two, three, and eight possibly cause maximum error ranges of 0.85–3.23 bpm, 1.28–4.76 bpm, and 3.83–11.76 bpm, respectively (*fs*: 100 Hz).

**Figure 11:**
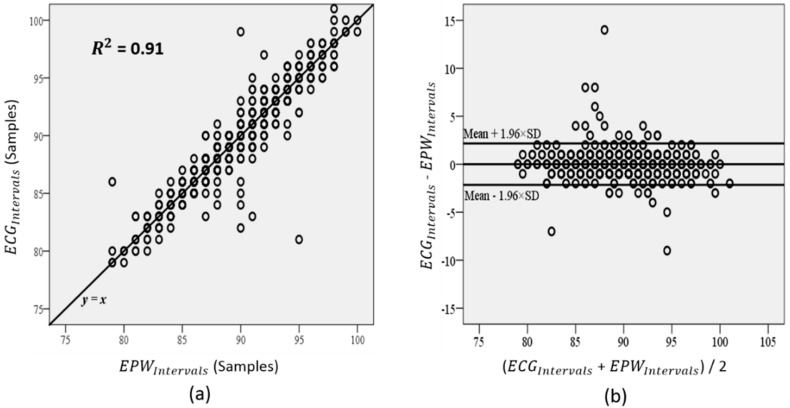
Agreement analysis results: (**a**) plot of identity; (**b**) Bland-Altman plot.

We examined two different plots: plot of identity and the Bland-Altman plot (Figure 11). In the plot of identity, the ECG_*Intervals*_ is plotted along the Y axis, and the EPW_*Intervals*_ is plotted along the X axis. The identity line is presented as a reference to compare two different intervals, which are desired to be identical under ideal conditions [29]. In the Bland-Altman plot, we created two variables: the average between ECG_*Intervals*_ and EPW_*Intervals*_, and the difference between two intervals. The difference is plotted along the Y axis, and the average is plotted along the X axis. Then, we drew 95% limits of agreement (average ± 1.96 SD of the difference) for comparison [29,33].

The identity line in Figure 11a is *y* = *x*; it represents a high level of agreement between EPW_*Intervals*_ and ECG_*Intervals*_. In the Bland-Altman plot (Figure 11b), the mean difference is 0.0065, the SD of the differences is 1.1011, and the 95% limits of agreement are −2.15 and 2.16. This figure shows that the difference does not have an increasing or decreasing trend, as the average increases. The estimated bias (mean difference) is very close to zero, and the random fluctuations (SD) around the bias are also narrow; thus, EPW_*Intervals*_ can be considered as being in high agreement with ECG_*Intervals*_.

## 6. Limitations

Despite the fact that our approach exhibits a competent performance, it has a drawback—this method is quite sensitive to the user’s motion. Because the piezoelectric sensor measures the pressure variance of the ear canal surface, if the user’s motions or activities become substantial, the pressure variance is affected and EPW peaks then become less distinct. Accordingly, our proposed method is limited to monitoring HR temporarily in the user’s stable status.

**Figure 12:**
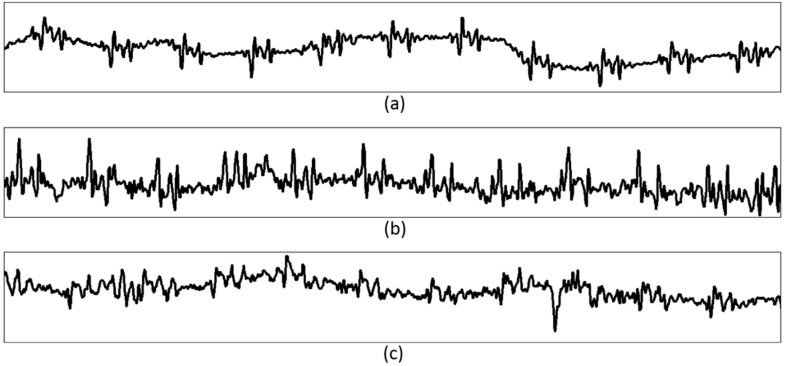
Examples of EPW waveform when the user is (**a**) walking, (**b**) running, (**c**) chewing gum.

However, as can be seen in Figure 12, which depicts examples of EPW waveforms that occur when the user is walking, running, and chewing gum, rough EPW peak signals for heartbeats are still recognizable. Thus, we believe that a more sophisticated and improved algorithm to detect EPW peaks will be required for continuous monitoring in normal daily life. Additional circuits or sensor devices will also be necessary to compensate for the user’s motion artifacts for more simple processing and extensive operation.

## 7. Conclusions

In this study, a novel approach was proposed for monitoring HR: the development of a wearable sensing device that is both highly accurate and comfortable to measure EPW. The scissor-shaped apparatus developed facilitates utilization of a small piezoelectric sensor and measurement of in-ear pressure variance with high wearability. The piezoelectric sensor was sufficiently sensitive to detect pressure waveforms from the ear canal surface. The hardware circuit module efficiently amplified EPW and filtered various noises, and the embedded algorithm properly detected EPW peaks via knowledge-based rules.

Among the new findings obtained in this study are the following: (1) HR can be accurately detected from the pressure variance of the ear canal surface; and (2) a piezoelectric sensor enables measurement as a part of a wearable sensor device. In addition, the validity study presented very promising results that prove that our approach is accurate and performs well.

We believe that this method could be used to monitor HR, and may also be utilized in diverse applications in the near future. One such instance could be to combine this method with a hearing aid. Hearing aids are already one of the most popular in-ear devices. The hearing impaired population, including the elderly, would be able to receive transparent and seamless vital sign monitoring benefits. There has been no known previous effort to produce this product, but reduction in the size and shape of the apparatus, and the circuit board could provide the key for the success of such a product as this.
